# Perceived stress level and risk of cancer incidence in a Japanese population: the Japan Public Health Center (JPHC)-based Prospective Study

**DOI:** 10.1038/s41598-017-13362-8

**Published:** 2017-10-11

**Authors:** Huan Song, Eiko Saito, Norie Sawada, Sarah K. Abe, Akihisa Hidaka, Taichi Shimazu, Taiki Yamaji, Atsushi Goto, Motoki Iwasaki, Shizuka Sasazuki, Weimin Ye, Manami Inoue, Shoichiro Tsugane

**Affiliations:** 1 Department of Medical Epidemiology and Biostatistics, Karolinska Institutet, Stockholm, Sweden; 2 AXA Department of Health and Human Security, Graduate School of Medicine, The University of Tokyo, 7-3-1 Hongo, Bunkyo-ku, Tokyo 113-0033 Japan; 3 Epidemiology and Prevention Group, Center for Public Health Sciences, National Cancer Center, 5-1-1 Tsukiji Chuo-ku, Tokyo, 104-0045 Japan; 4 Division of Cancer Statistics Integration, Center for Cancer Control & Information Services, National Cancer Center, 5-1-1 Tsukiji Chuo-ku, Tokyo, 104-0045 Japan; 5 Department of Global Health Policy, Graduate School of Medicine, The University of Tokyo, 7-3-1 Hongo, Bunkyo-ku, Tokyo 113-0033 Japan

## Abstract

Evidence regarding stress as a risk factor for cancer onset is inconsistent. In this study, based on the Japan Public Health Center-based Prospective Study, we enrolled 101,708 participants aged 40–69 years from 1990–1994. The self-reported perceived stress level was collected at baseline and updated through 5-year follow-up. The association between perceived stress and cancer risk was measured by Cox proportional hazards regression model, adjusted for all known confounders. During follow-up (mean = 17.8 years), we identified 17,161 cancer cases. We found no association between baseline perceived stress level and cancer incidence. However, by taking account of the dynamic changes in perceived stress, time-varying analyses revealed a slightly (4–6%) increased overall cancer risk for subjects under elevated perceived stress levels compared to the ‘low stress level’ group. Analyses concerning long-term perceived stress level showed that individuals with constantly high perceived stress level had an 11% (95% confidence interval 1–22%) excess risk for cancer compared to subjects with persistently low stress levels. This association was confined to men (20% excess risk), and was particularly strong among smokers, alcohol drinkers, obese subjects, and subjects without family history of cancer. Therefore, we concluded high perceived stress level might contribute to excess overall cancer incidence among men.

## Introduction

Previous studies have indicated apparent links between stress and many adverse health outcomes (e.g. cardiovascular disease^1,2^, diabetes^3^). However, evidence regarding stress as a risk factor for cancer onset is inconsistent^4–10^. These mixed results may be partly attributable to practical differences in stress measurement (in addition to subjective questioning about perceived stress level, common objective indictors for psychological stress include stress-prone personality, coping strategy, and social support); possible methodological weaknesses (cross-sectional/retrospective study design, or small sample size); difficulties in completing long-term prospective follow-up; and the fact that cancer incidence is influenced by many other factors. Moreover, although exposure to chronic stress, rather than temporary stress, has long been considered a major cause of health problems^11,12^ via long-term impacts on biological processes or behavioral patterns, studies providing effective assessment of the health impact of long-term stress levels are scarce.

Here, we aimed to more accurately evaluate the association between self-reported perceived stress level, as well as changes over a 5-year period, and the risk of a future cancer diagnosis among the Japanese population. We used data from a large prospective population-based cohort study in Japan with repeated stress data from both baseline and 5 years’ follow-up, sufficient information on other cancer-related risk factors, and a surveillance period for cancer occurrence of up to 22 years. Our research hypothesis is, irrespective of the variety of stressor, individuals having a feeling of under high level of daily stress are more vulnerable to cancers, compared to subjects without such feeling.

## Methods

### Study population

This project was based on data from the Japan Public Health Center-based Prospective Study (JPHC Study), which enrolled 140,420 residents aged 40–69 years registered at 11 public health center (PHC) areas nationwide from 1990–1994. These residents were also invited to participate in 5- and 10-year follow-ups after the initial survey. Information including personal and family medical history, lifestyle, psychosocial factors, and anthropometric indexes was collected with a self-administered questionnaire. Details of this study have been described previously^13^. Briefly, response rate for the initial investigation, 5-year follow-up, and 10-year follow-up was 81%, 74%, and 71%, respectively. The JPHC Study, including all methods described in the current study, has been approved by the Institutional Review Board of the National Cancer Center (approval number: 2001–021) and the University of Tokyo (approval number: 10508), with reference to relevant ethical guidelines for medical research in Japan. Informed consent was obtained from each participant implicitly when they completed the baseline questionnaire, in which the purpose of the study and follow-up methods were well described and explained. Written information on the study was mailed to each participant, and is published on the study web site (http://epi.ncc.go.jp/jphc).

### Study design

**Figure 1:**
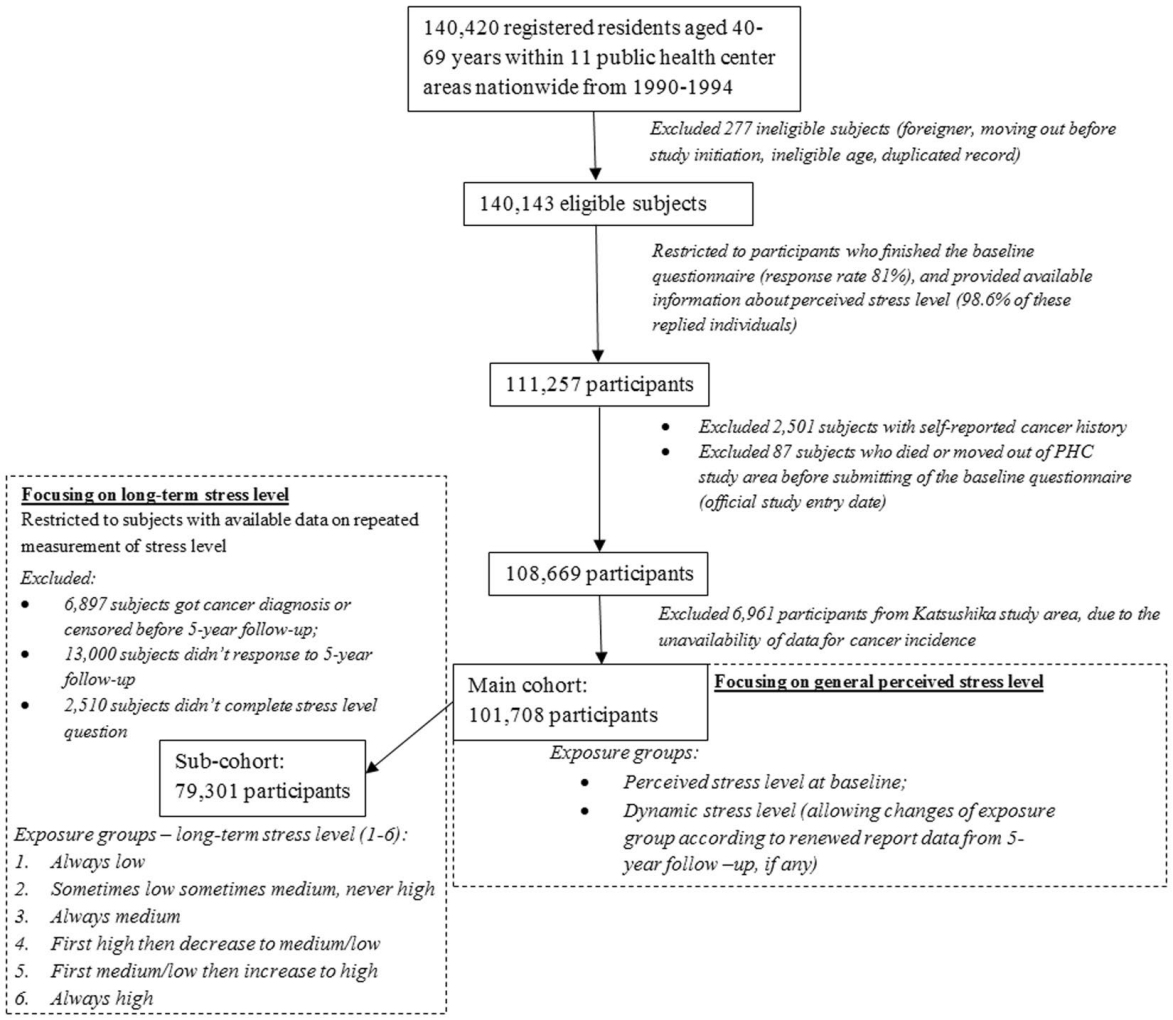
Study design.

We conducted the present study among 111,257 eligible subjects with complete information on perceived stress level at baseline (Fig. 1). Subjects with a self-reported cancer history (n = 2,501), or who died or moved out their PHC area (n = 87) before baseline enrollment (study entry, defined as submission date of the baseline questionnaire) were excluded. We further excluded 6,961 subjects from one PHC (Katsushika) due to unavailability of data for cancer incidence in this area, leaving 101,708 participants for further analysis. All individuals were followed from study entry until first cancer diagnosis, moving out of the study area, death, or end of follow-up (31 December 2012), whichever occurred first.

#### Perceived stress level and its change over time

Perceived stress level was determined by the response to one item in the questionnaire— ‘How much stress do you have in your daily life’? We considered that perceived stress level was low if the participants reported ‘a little’ to this question, and medium and high if they reported ‘average’ and ‘a lot’, respectively.

We used baseline and 5-year follow-up data to measure dynamic stress level, as the questionnaire for the 10-year follow-up did not include this item. In addition, based on our concern that subjects who had ever experienced high stress levels actually held a different cancer risk to those who had never experienced a period of high stress, we established a sub-cohort which was limited to participants with available repeated stress assessment data from the 5-year follow-up (Fig. 1, n = 79,301) and categorized the subjects into six groups according to their long-term perceived stress level (from low to high): 1 = always low stress level; 2 = sometimes low, sometimes medium stress level; 3 = always medium stress level; 4 = high at baseline then decreasing to low/medium stress level at 5-year follow-up; 5 = low/medium at baseline then increasing to high stress level at 5-year follow-up; and 6 = always high stress level.

#### Cancer case ascertainment

We identified cancer cases through data linkage with cancer registers, or/and notification from local hospitals in the study areas. Cancer sites were coded according to the International Classification of Disease for Oncology, Third Edition (ICD-O-3)^14^. Furthermore, since perceived stress might affect the time of presentation (i.e. the time the patient chose to visit the doctor with symptoms), we specified incident cancer in sub-analyses as ‘screening-detected’ cancer or ‘localized’ or ‘non-localized’ cancer (for these detected through hospital visits). Localized cancer was defined as having no regional lymph node or distant organ metastases at the time of diagnosis^6^.

### Statistical analysis

Among all eligible participants, we evaluated the association between perceived stress level and cancer risk using hazard ratios (HRs) with 95% confidence intervals (CIs), derived from a Cox proportional hazards regression model. First, we grouped subjects simply by their stress level at baseline. Then, to allow for a change in exposure group according to the re-estimated stress data from the 5-year follow-up (if any), we split the dataset and applied the counting process model with a robust sandwich estimate for the time-varying analysis. Namely, subjects having different stress levels between baseline and follow-up contributed their person-time to baseline stress groups before the completion date of the 5-year follow-up questionnaire, and to re-estimated stress groups thereafter. We always used attained age as the underlying time scale. The simple model was adjusted for gender and stratified by PHC area. Furthermore, we involved other psychologically related covariates (i.e., perceived level of life enjoyment [low, medium, or high], sleep hours [≤6, 7–8, or ≥9], type A behavioral pattern [low, medium, high, or very high index])^15^, as well as other known main cancer risk factors (e.g. smoking status [never, former, or current], alcohol intake level [never, rare, <23, 23–46, 47–69, 70–92, or >92 g/day], BMI [<18.5, 18.5–25, 25–30, or >30 kg/m^2^], occupation [professional worker, sales clerks, farmer, other, or unemployed], physical activity [almost none, 1–3 times/month, 1–2 times/week, 3–4 times/week, or almost every day], living arrangement [living alone/living with others], fruit and vegetable consumption [by quartile], and family history of cancer [yes/no]) in multivariate models. Men and women were then analyzed separately. Further, overall cancer incidence and relative risks for different types of cancer (esophageal, gastric, colon, rectal, liver, pancreatic, lung, breast, and prostate cancer) were evaluated separately. P values for linear trends was calculated by assigning ordinal variables for gradually increased stress levels and entering the number as a continuous variable into the model. We checked the proportional hazards assumption graphically and by Schoenfeld’s partial residuals, and found no indication of violation.

In addition, with a specific interest in the predictive value of long-term perceived stress level, we further restricted our analyses to subjects with available repeated stress assessment data. The associations between long-term stress level (in 6 categories) and cancer incidence were assessed by Cox regression models after controlling for the covariates stated above. To detect possible effect modification, we performed subgroup analyses by gender, family history of cancer, alcohol intake level, smoking status, and BMI level. The significance of the interaction was statistically checked by incorporating the cross-product terms of stress level and subgrouping variables into the models.

We then repeated all the above analyses for cancers detected through screening or hospital visits (further specified as localized or non-localized cancer at diagnosis). Also, to avoid perceived stress levels that were influenced by current cancer disease (early symptoms of undiagnosed cancer disease at the time of interview) which would bias our results, we performed sensitivity analyses by excluding cancer cases diagnosed in the first two years of follow-up (or for analyses of long-term stress level, diagnosed in the first two years after the 5-year follow-up). Additional sensitivity analyses, such as the exclusion of participants with severe disease (e.g. diabetes, cardiovascular disease, hepatitis) and those with extremely long working hours (≥10 hours)^16^, were also conducted.

A p value less than 0.05 was considered statistically significant. All analyses were conducted with SAS statistical software, version 9.4 (Cary, NC).

### Patient involvement

No patients were involved in setting the research question or the outcome measures, nor were they involved in the design and implementation of the study. There are no plans to involve patients in dissemination. The study findings will be disseminated to study participants through physician newsletters and the mass media.

## Results

Table 1 illustrates the characteristics of all participants, as well as subjects by stress level group at baseline. Participants had a mean follow-up time of 17.8 years, corresponding to an accrued 1,805,828 person years at risk. Mean age at study entry was 53 years. Younger participants seemed to have a higher stress level than older ones. Although the whole cohort almost reached gender equity (female: male = 1.05:1), more men were present in the highest stress level group (52.8%). In addition, we found in general that highly stressful persons tended to have more unhealthy lifestyle habits—i.e., they were more likely to be smokers, alcohol drinkers, and physically inactive, in comparison to subjects with lower stress levels. Typical type A behavior pattern, identified through a high or very high overall index for Type A behaviors^15^, was prevalent (about 49%) among subjects with high stress levels. Moreover, stress level was inversely associated with the length of sleeping hours, as well as life enjoyment level.

**Table 1:** Characteristics of participants, overall and stratified by perceived level of stress at baseline.

	Overall	Perceived stress level at baseline		
		Low	Medium	High
Total n	101,708	16,167	64,180	21,361
Age, mean ± S.D	52.8 ± 8.1	55.0 ± 8.6	53.2 ± 8.0	50.0 ± 7.0
Years of follow-up, mean ± S.D	17.8 ± 5.9	17.3 ± 5.8	17.8 ± 5.8	17.8 ± 6.0
Sex, n (%) of males	48588 (47.8)	7161 (44.3)	30059 (46.8)	11368 (53.2)
Body mass index level				
<18.5 kg/m^2^	4369 (4.30)	690 (4.27)	2768 (4.31)	911 (4.26)
18.5–25 kg/m^2^	69579 (68.4)	10836 (67.0)	44018 (68.6)	14725 (68.9)
25–30 kg/m^2^	25081 (24.7)	4142 (25.6)	15787 (24.6)	5152 (24.1)
>30 kg/m^2^	2643 (2.60)	496 (3.07)	1586 (2.47)	561 (2.63)
Missing	36 (0.04)	3 (0.02)	21 (0.03)	12 (0.06)
Alcohol consumption				
Never	51242 (50.4)	8886 (55.0)	33263 (51.8)	9093 (42.6)
Rare (<1 time/week)	9543 (9.38)	1308 (8.09)	6004 (9.35)	2231 (10.4)
<23 g/day	7743 (7.61)	1118 (6.92)	4563 (7.11)	2062 (9.65)
23–46 g/day	12001 (11.8)	1720 (10.6)	7361 (11.5)	2920 (13.7)
47–69 g/day	8751 (8.60)	1178 (7.29)	5366 (8.36)	2207 (10.3)
70–92 g/day	6062 (5.96)	898 (5.55)	3808 (5.93)	1356 (6.35)
>92 g/day	4822 (4.74)	767 (4.74)	2783 (4.34)	1272 (5.95)
Missing	1544 (1.52)	292 (1.81)	1032 (1.61)	220 (1.03)
Smoking status				
Non-smoker	60033 (59.0)	10121 (62.6)	38691 (60.3)	11221 (52.5)
Former smoker	12285 (12.1)	1940 (12.0)	7559 (11.8)	2786 (13.0)
Current smoker	28908 (28.4)	4037 (25.0)	17597 (27.4)	7274 (34.1)
Missing	482 (0.47)	69 (0.43)	333 (0.52)	80 (0.37)
Fruit intake amount				
1st quartile (lowest)	25658 (25.2)	4359 (27.0)	15858 (24.7)	5441 (25.5)
2nd quartile	22993 (22.6)	3559 (22.0)	14510 (22.6)	4924 (23.1)
3rd quartile	23966 (23.6)	4079 (25.2)	15245 (23.8)	4642 (21.7)
4th quartile (highest)	25175 (24.8)	3575 (22.1)	16242 (25.3)	5358 (25.1)
Missing	3916 (3.85)	595 (3.68)	2325 (3.62)	996 (4.66)
Vegetable intake amount				
1st quartile (lowest)	27077 (26.6)	4894 (30.3)	16700 (26.0)	5483 (25.7)
2nd quartile	26416 (26.0)	4933 (30.5)	16357 (25.5)	5126 (24.0)
3rd quartile	15419 (15.2)	2397 (14.8)	9758 (15.2)	3264 (15.3)
4th quartile (highest)	32572 (32.0)	3909 (24.2)	21228 (33.1)	7435 (34.8)
Missing	224 (0.22)	34 (0.21)	137 (0.21)	53 (0.25)
Occupation				
Professional or office workers	24794 (24.4)	3150 (19.5)	13795 (21.5)	7849 (36.7)
Sales clerks or others	22959 (22.6)	2954 (18.3)	14159 (22.1)	5846 (27.4)
Famers	21382 (21.0)	4130 (25.6)	14647 (22.8)	2605 (12.2)
Other	10341 (10.2)	1036 (6.41)	7078 (11.0)	2227 (10.4)
Unemployed incl. housewives	21263 (20.9)	4768 (29.5)	13808 (21.5)	2687 (12.6)
Missing	969 (0.95)	129 (0.80)	693 (1.08)	147 (0.69)
Physical activity				
Almost none	70571 (69.4)	10843 (67.1)	44735 (69.7)	14993 (70.2)
1–3 times/month	11359 (11.2)	1463 (9.05)	7065 (11.0)	2831 (13.3)
1–2 times/week	9397 (9.24)	1634 (10.1)	5820 (9.07)	1943 (9.10)
3–4 times/week	4322 (4.25)	909 (5.62)	2699 (4.21)	714 (3.34)
Almost everyday	4991 (4.91)	1146 (7.09)	3121 (4.86)	724 (3.39)
Missing	1068 (1.05)	172 (1.06)	740 (1.15)	156 (0.73)
Family history of cancer (parents/siblings)				
No	79744 (78.4)	12774 (79.0)	50891 (79.3)	16079 (75.3)
Yes	21964 (21.6)	3393 (21.0)	13289 (20.7)	5282 (24.7)
Living arrangement: living alone				
No	97197 (95.6)	15201 (94.0)	61469 (95.8)	20527 (96.1)
Yes	4170 (4.10)	919 (5.68)	2455 (3.83)	796 (3.73)
Missing	341 (0.34)	47 (0.29)	256 (0.40)	38 (0.18)
Level of life enjoyment				
Low	8707 (8.56)	670 (4.14)	3755 (5.85)	4282 (20.1)
Medium	48644 (47.8)	4517 (27.9)	33009 (51.4)	11118 (52.1)
High	41770 (41.1)	9936 (61.5)	26159 (40.8)	5675 (26.6)
Missing	2587 (2.54)	1044 (6.46)	1257 (1.96)	286 (1.34)
Sleep hours				
≤6 hours	4516 (4.44)	567 (3.51)	2464 (3.84)	1485 (6.95)
7–8 hours	86767 (85.3)	13071 (80.9)	55289 (86.2)	18407 (86.2)
≥9 hours	8181 (8.04)	1746 (10.8)	5227 (8.14)	1208 (5.66)
Missing	2244 (2.21)	783 (4.84)	1200 (1.87)	261 (1.22)
Type A behavior pattern				
Low overall index	22338 (22.0)	4534 (28.0)	13529 (21.1)	4275 (20.0)
Medium overall index	36798 (36.2)	5192 (32.1)	25937 (40.4)	5669 (26.5)
High overall index	15041 (14.8)	2057 (12.7)	9380 (14.6)	3604 (16.9)
Very high overall index	19889 (19.6)	2339 (14.5)	10773 (16.8)	6777 (31.7)
Missing	7642 (7.51)	2045 (12.7)	4561 (7.11)	1036 (4.85)

### Association between perceived stress level at baseline and cancer incidence

During follow-up, 17,161 participants were diagnosed with cancer. Regarding baseline perceived stress level, we found a significant but trivial differences (6% at the most) in cancer incidence for subjects among different exposure groups, using a simply adjusted regression model (Table 2). However, this effect disappeared when more covariates were added into the model. Similar findings were noted for both men and women.

**Table 2:** Hazard ratios (HR) and 95% confidence intervals (CIs) for cancer incidence among participants with different perceived stress levels.

Perceived stress level	HR (95% CI)		
	Model 1^*^	Model 2^†^	Model 3^£^
*Stress level at baseline*			
**All**			
Low	Reference	Reference	Reference
Medium	1.02 (0.98–1.07)	1.01 (0.97–1.06)	1.01 (0.97–1.06)
High	1.06 (1.00–1.11)	1.05 (0.99–1.10)	1.02 (0.96–1.07)
*P for trend*	0.0472	0.1042	0.5833
**Males**			
Low	Reference	Reference	Reference
Medium	1.01 (0.95–1.06)	1.00 (0.95–1.06)	1.01 (0.95–1.06)
High	1.05 (0.98–1.12)	1.03 (0.96–1.11)	1.01 (0.95–1.09)
*P for trend*	0.1688	0.3435	0.6738
**Females**			
Low	Reference	Reference	Reference
Medium	1.05 (0.98–1.12)	1.04 (0.97–1.11)	1.04 (0.97–1.12)
High	1.11 (1.02–1.21)	1.09 (1.00–1.20)	1.08 (0.98–1.18)
*P for trend*	0.0185	0.0464	0.1075
*Dynamic stress level (allowing change of exposure group according to data from 5-year follow-up)*			
**All**			
Low	Reference	Reference	Reference
Medium	1.05 (1.01–1.09)	1.05 (1.01–1.09)	1.04 (1.01–1.09)
High	1.09 (1.03–1.14)	1.08 (1.02–1.13)	1.06 (1.00–1.11)
*P for trend*	0.0009	0.0049	0.0482
**Males**			
Low	Reference	Reference	Reference
Medium	1.08 (1.03–1.14)	1.08 (1.03–1.13)	1.07 (1.02–1.13)
High	1.14 (1.07–1.22)	1.13 (1.06–1.21)	1.10 (1.03–1.18)
*P for trend*	<0.0001	0.0001	0.0020
**Females**			
Low	Reference	Reference	Reference
Medium	1.02 (0.96–1.08)	1.02 (0.96–1.08)	1.01 (0.95–1.08)
High	1.05 (0.96–1.14)	1.05 (0.96–1.14)	1.02 (0.93–1.10)
*P for trend*	0.2810	0.4666	0.7001

^*^Estimated by a simply adjusted Cox proportional hazards regression model (using attained age as underlying time scale): adjusted for sex and stratified by study areas.

^†^Estimated by semi-adjusted Cox proportional hazards regression model (using attained age as underlying time scale): adjusted for sex and psychological-related factors (sleeping hours, perceived level of enjoyment, type A behavior pattern), and stratified by study areas.

^£^Estimated by fully-adjusted Cox proportional hazards regression model (using attained age as underlying time scale), adjusted for sex, psychologically related factors, and other known risk factors for cancer (body mass index, smoking status, alcohol consumption, fruit/vegetable intake, living arrangement, physical activity, occupation, family history of cancer), and stratified by study area.

### Association between dynamic perceived stress level and cancer incidence

**Figure 2:**
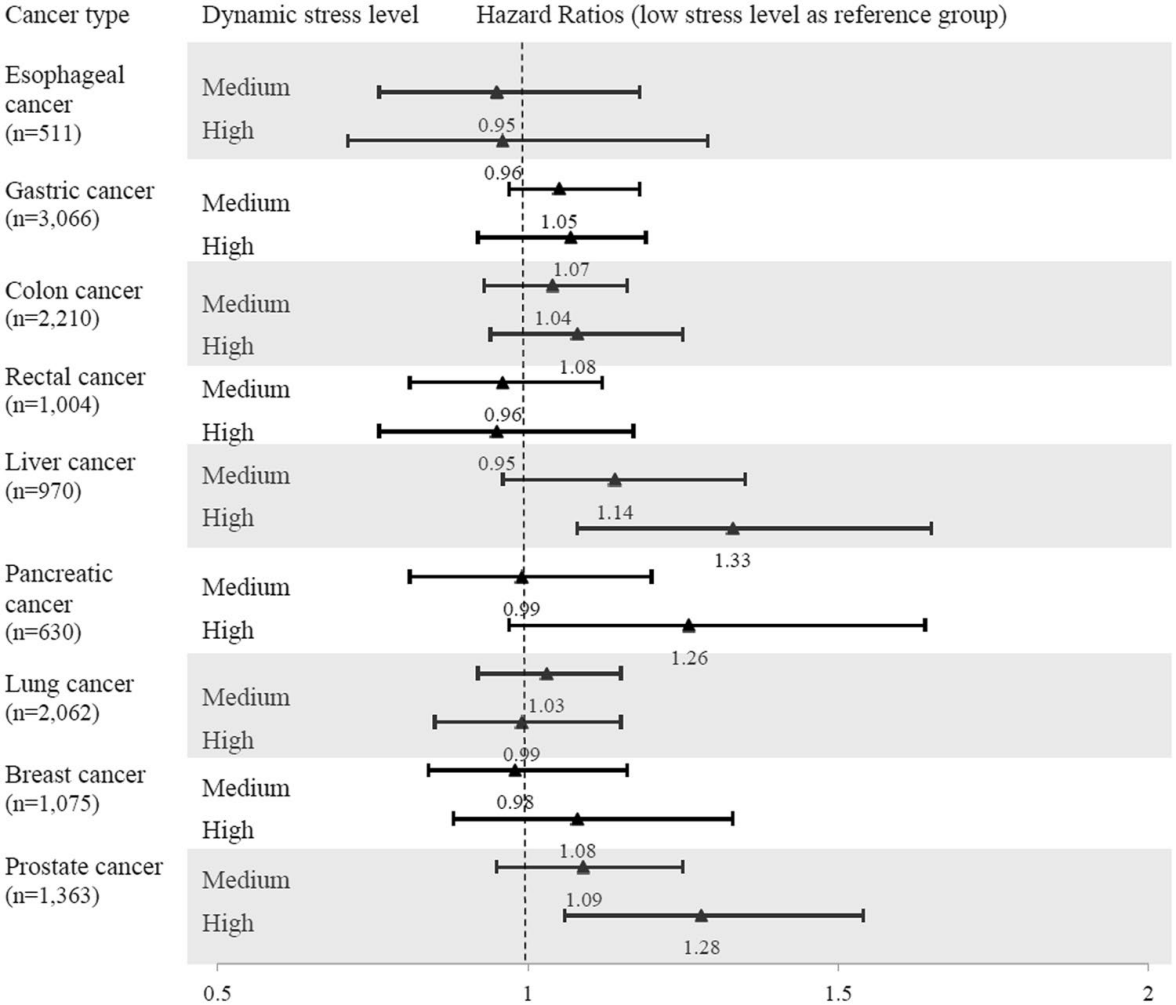
Hazard ratios (HR) and 95% confidence intervals (CIs) for different type cancer incidence among participants with different dynamic stress level.

Longitudinal data on perceived stress level were taken into account by implementing a time-varying analysis. In general, after controlling for all available confounders, we observed a slight but significant increase in the risk of having a cancer diagnosis for subjects under either a medium or high level of stress, compared to the reference group (‘low stress level’), with multivariable adjusted HRs of 1.04 (95% CI 1.01–1.09) for the medium stress level group and 1.06 (95% CI 1.00–1.11) for the high stress group, showing a clear trend to increase (Table 2, p for trend = 0.048). On sub-grouping by gender, we found the observed associations were exaggerated among men but attenuated to an insignificant level among women. Analysis by type of cancer (Fig. 2) indicated that perceived stress level was most commonly associated with liver cancer and prostate cancer.

### Association between long-term perceived stress level and cancer incidence

To explore long-term perceived stress, we restricted our further analyses to the 79,301 subjects who had repeated data on stress (78% of all participants in the main analysis). Comparison of the characteristics of participants with and without repeated stress assessment (still at risk after 5 years of baseline survey, n = 15,510) revealed only small differences between these two subgroups (data not shown). Adjusted HRs and 95% CIs for cancer incidence related to long-term perceived stress level are summarized in Table 3. Overall, the relative risk of cancer increased with higher long-term stress levels (p for trend = 0.0002)—individuals who had a constantly high perceived stress level had an 11% excess risk for cancer compared to those with persistently low stress levels. This association was confined to men, where the HR for highly stressed subjects (persistently high or became high from 5 years after baseline survey) was approximately 1.20. For women, an elevated point estimate for HR was observed only among subjects with a persistently high stress level.

**Table 3:** Hazard ratios (HRs) and 95% confidence intervals (CIs) for cancer incidence in relation to long-term perceived stress level, determined by self-reported data from both baseline and 5-year follow-up.

Long-term perceived stress level	All (n = 79,301)		Males (n = 36,512)		Females (n = 42,789)	
	Observed cases	Fully adjusted HR (95% CI)^*^	Observed cases	Fully adjusted HR (95% CI)^*^	Observed cases	Fully adjusted HR (95% CI)^*^
Always low	944	Reference	560	Reference	384	Reference
Low or medium (never high)	2523	0.98 (0.90–1.05)	1554	1.01 (0.92–1.12)	969	0.93 (0.83–1.05)
Always medium	5636	1.00 (0.93–1.07)	3393	1.05 (0.96–1.15)	2243	0.94 (0.84–1.05)
First high then low/medium	1438	1.04 (0.96–1.14)	924	1.10 (0.99–1.23)	514	1.00 (0.87–1.15)
First low/medium then high	982	1.09 (1.00–1.20)	578	1.20 (1.07–1.35)	404	0.99 (0.85–1.14)
Always high	963	1.11 (1.01–1.22)	598	1.19 (1.05–1.34)	365	1.07 (0.92–1.25)
*P for trend*		0.0002		<0.0001		0.1227

^*^Estimated by a fully adjusted Cox proportional hazards regression model (using attained age as underlying time scale), adjusted for sex (only for all), psychologically related factors, and other known risk factors for cancer (body mass index, smoking status, alcohol consumption, fruit/vegetable intake, living arrangement, physical activity, occupation, family history of cancer), and stratified by study area.

Stratification analyses (Table 4) revealed that an association between long-term perceived stress level and cancer incidence was valid only for subjects without a genetic background (representing as not having a family history of cancer). Also, we found that long-term perceived stress level was more likely to be relevant for subjects with a drinking or smoking habit compared to non-drinkers or non-smokers. Further, in terms of BMI level, we observed similar trends as in the main analysis for underweight, normal, overweight subjects. For obese participants, while no clear dose-response relationship was seen (p for trend = 0.1137), elevated long-term stress level was generally associated with a 20% to 77% excess risk for cancer.

**Table 4:** Hazard ratios (HRs) and 95% confidence intervals (CIs) for cancer incidence in relation to long-term perceived stress level, stratified by family history of cancer, alcohol intake level, smoking status, and body mass index (BMI) level.

Long-term perceived stress level	Fully adjusted HR (95% CI)^*^							*P for interaction*
***Stratified by family history of cancer***	No family history of cancer, n = 61,837			Having family history of cancer, n = 17,464				
Always low	Reference			Reference				0.37
Low or medium (never high)	0.97 (0.89–1.06)			0.99 (0.85–1.16)				
Always medium	1.01 (0.93–1.09)			0.97 (0.84–1.12)				
First high then low/medium	1.06 (0.96–1.17)			0.99 (0.83–1.17)				
First low/medium then high	1.14 (1.03–1.27)			0.95 (0.78–1.15)				
Always high	1.11 (1.00–1.24)			1.11 (0.92–1.34)				
*P for trend*	0.0001			0.4187				
*Stratified by alcohol intake level*	Non-drinker, n = 40,741		Low-alcohol drinker (<46d/day), n = 22,618			High-alcohol drinker (≥46d/day), n = 14,862		
Always low	Reference		Reference			Reference		0.29
Low or medium (never high)	0.90 (0.80–1.00)		1.09 (0.94–1.27)			1.06 (0.91–1.25)		
Always medium	0.92 (0.83–1.02)		1.10 (0.96–1.27)			1.12 (0.97–1.30)		
First high then low/medium	0.93 (0.82–1.06)		1.17 (1.00–1.39)			1.17 (0.98–1.39)		
First low/medium then high	1.00 (0.87–1.14)		1.14 (0.95–1.36)			1.30 (1.08–1.57)		
Always high	1.01 (0.87–1.17)		1.21 (1.01–1.45)			1.27 (1.06–1.54)		
*P for trend*	0.1512		0.0367			0.0004		
*Stratified by smoking status*	Non-smoker, n = 48,503		Former smoker, n = 9,391			Current smoker, n = 21,071		
Always low	Reference		Reference			Reference		0.60
Low or medium (never high)	0.92 (0.82–1.02)		1.07 (0.88–1.30)			1.03 (0.91–1.18)		
Always medium	0.93 (0.84–1.03)		1.10 (0.92–1.33)			1.05 (0.93–1.19)		
First high then low/medium	0.95 (0.84–1.08)		1.19 (0.96–1.49)			1.14 (0.99–1.32)		
First low/medium then high	1.00 (0.88–1.14)		1.24 (0.97–1.58)			1.20 (1.03–1.40)		
Always high	1.07 (0.93–1.23)		1.15 (0.90–1.47)			1.19 (1.02–1.40)		
*P for trend*	0.0715		0.0896			0.001		
*Stratified by BMI level*	Underweight, n = 3,147	Normal, n = 54,463			Overweight, n = 19,657		Obesity, n = 2,021	
Always low	Reference	Reference			Reference		Reference	0.98
Low or medium (never high)	0.83 (0.58–1.20)	0.96 (0.88–1.05)			0.98 (0.85–1.14)		1.47 (0.92–2.36)	
Always medium	0.92 (0.65–1.29)	0.98 (0.90–1.07)			1.01 (0.88–1.17)		1.48 (0.94–2.32)	
First high then low/medium	1.08 (0.71–1.64)	1.02 (0.92–1.14)			1.07 (0.90–1.27)		1.19 (0.67–2.10)	
First low/medium then high	1.08 (0.71–1.64)	1.04 (0.93–1.17)			1.16 (0.97–1.39)		1.77 (1.02–3.09)	
Always high	1.26 (0.80–2.01)	1.08 (0.96–1.21)			1.11 (0.92–1.34)		1.71 (0.96–3.06)	
*P for trend*	0.063	0.0206			0.0241		0.1137	

^*^Estimated by a fully adjusted Cox proportional hazards regression model (using attained age as underlying time scale), adjusted for sex, psychological-related factors, and other known risk factors for cancer (body mass index, smoking status, alcohol consumption, fruit/vegetable intake, living arrangement, physical activity, occupation, family history of cancer), and stratified by study area. For each stratification analysis, the stratifying factor was not used for adjustment.

By specifying the timing of cancer diagnosis (screening-detected cancer [n = 2,752], or localized [n = 5,639] or non-localized [n = 8,770] cancer at the time of diagnosis), we found that the perceived stress level was likely to be relevant to screening-detected cancer incidence and localized cancer incidence (Supplementary Table 1). In contrast, for non-localized cancers that were diagnosed through hospital visits, no such linkage was found. Sensitivity analyses that excluded cancer cases occurring during the first two years of follow-up, excluding participants with a self-reported severe disease history, or excluding those with long working hours, did not change the findings described above.

## Discussion

With supporting evidence from experimental animal studies since 1970s^17–19^, the question of whether stress increases cancer incidence has been a focus for epidemiological research. With different strategies for stress measurement and varied study designs, previous investigations have provided disparate, and therefore only suggestive, evidence on this issue^20–22^. Conceivably, given that stress level can change over time^23^, longitudinal data on stress are naturally superior to a single baseline measure; such data, however, are scarce. To our knowledge, this is the first large population-based cohort study to describe the association between repeated measures of perceived stress level and cancer incidence. Although we found no evidence for an association between perceived stress level at baseline and the overall risk of cancer, our results indicate that both dynamically and persistently high perceived stress were significantly linked to an increased overall cancer incidence, and particularly to an elevated incidence of liver and prostate cancer. The observed association was confined to men, and was particularly clear among subjects without an inherited genetic background (no family history of cancer), and those having high-risk behavior patterns (e.g. smokers or alcohol drinkers). In addition, given that this stress-related increase in cancer incidence only existed for screening-detected cancer and localized cancer that was diagnosed through hospital visits, we hypothesize that either stress-induced cancers are less invasive or aggressive than cancers initiated via other mechanisms (not supported by data yet), or that a high level of perceived stress could lead to prompt presentation of the presence of early symptoms.

Previous studies on the relationship between stress or stress-related psychosocial factors and cancer incidence are considered to have been of generally poor quality^10^. In the present study, we found no excess cancer risk in relation to a high baseline perceived stress level. This is consistent with findings from other large-scale prospective cohorts in which daily stress was considered an exposure^7,24^. Although contrasting results have also been reported^21,25,26^, given the differences in stress components examined, follow-up period, and study population, the heterogeneity of these outcomes does not necessarily invalidate our findings.

Our attempt to use repeat measures of perceived stress for association assessment is new. Without similar data for comparison, our results indicated a small but significant effect of perceived stress on overall cancer risk: namely, a high stress level captured by repeated measures was linked with a 6% increase in overall cancer incidence compared to those with low perceived stress. Such excess became more pronounced (11%) when we specified the changing pattern of stress within a 5-year duration (comparing subjects with persistently high perceived stress to those with persistently low stress). Our finding that perceived stress was more relevant for men than women is also consistent with prior reports; indeed, studies focusing on the detrimental effects of stress among women constantly showed null results^10,24,27,28^. A possible explanation for this phenomenon is that Japanese males are differed from females with regard to many aspects of lifestyle (e.g. occupation type, smoking and drinking status); and stressed men are more prone than stressed women to change their lifestyle (start smoking or heavy drinking) or continue their unhealthy habits to release their stress. Alternatively, compared to women, men might have greater reactivity of the hypothalamic-pituitary-adrenal axis when exposed to psychological stress^29^.

Our finding that liver cancer is one of the most stress-related cancer types fits the theory that stress promotes carcinogenesis through impaired immune surveillance (liver cancer is considered an immunogenic cancer^30^). Moreover, other data from both clinical and animal studies have also linked stress with the evolution of various liver diseases (viral hepatitis, cirrhosis and hepatocellular carcinoma)^31^. Regarding the relationship between stress and prostate cancer, further studies are needed to confirm our results. Evidence from epidemiological studies is mixed^9,32^, although some researchers have pointed out the neurochemical noradrenaline, a primary stress hormone that promotes the growth of prostate tumors in its early phase^33^, which in turn provides a potential mechanism to explain such an association. A link between other types of cancers, especially breast^4^ and lung cancer^25^, has also been suspected to be linked with stress-related factors, albeit that results for these in our present analysis were null.

Given that only a moderate association was observed between perceived stress level and cancer, the differential effects by family history of cancer are understandable. In the presence of a genetic predisposition, the influence of stress might become negligible. The clearer associations observed among smokers, alcohol drinkers, and obese persons have been rarely described in previous papers. This also raises the question whether perceived stress linked directly cancer incidence or, perceived stress changed some life style and then increased cancer risk. Although significant interaction effects between these high-risk behaviors and perceived stress level were absent in our analyses, our results suggest the possibility that other physical risk factors are indispensable, or at least play a critical role, in the pathogenesis of stress-induced cancer^34^.

In addition to its fairly large sample size, prospective study design, high response rate, and sufficiently long follow-up period, the major strength of our study is the completeness of stress data at baseline, as well as the availability of repeat measures on perceived stress level for most of the baseline participants (78%). Consequently, high stress occurring after baseline was detectable; and we obtained a unique chance to specify the stress levels by their changing patterns over time (partly captured chronic stressful persons). Moreover, the similar results we obtained using two approaches to analyze longitudinal stress data make it unlikely that the observed associations were merely due to chance.

Given that perceived stress was assessed by a single question during the surveys without validation against any extensive stress scale (such as perceived stress scale^35^), the observed effects might have been influenced by possible misclassification. However, recent investigations have demonstrated sufficient reliability and validity of single-item measures for stress or stress-related factors^36–39^. The correlations detected between perceived stress level and other psychologically related factors (sleeping hours, life enjoyment) in our study further implied its effectiveness. Moreover, although a single-item measure is acknowledged to be more vulnerable to random measurement error than multiple-item measure^40^, our repeated measures of stress might have helped avoid severe deviation caused by this problem. No available information about the mental condition (e.g. depression, anxiety) of participants, either preceding or after the baseline survey, limited our insights into the modification effect of mental disorders on the studied association. This topic therefore requires further discussion in future studies.

Due to the relatively small effect sizes we observed in current results, despite adequate adjustment of confounders, we cannot rule out the possibility that residual confounding or unmeasured confounding from unknown confounders has biased our results. Additionally, geographical differences in lifestyle and cancer incidence mean that the generalizability of our results to other populations remains uncertain.

In conclusion, our investigation shows that dynamically or persistently high perceived stress levels might contribute to a 10–20% excess risk of developing cancer among men. However, a single measure of perceived stress exposure seems incapable of showing such an association.

## Electronic supplementary material

Supplementary Table
