# Supplementary material for: Perceived stress level and risk of cancer incidence in a Japanese population: the Japan Public Health Center (JPHC)-based Prospective Study
Source: Sci Rep. 2017 Oct 11;7:12964. doi: 10.1038/s41598-017-13362-8 (PMC5636815; doi:10.1038/s41598-017-13362-8)
Supplement: Supplementary file 1 — Supplementary Table [file 41598_2017_13362_MOESM1_ESM.doc]

**Perceived stress level and risk of cancer incidence in a Japanese population: the Japan Public Health Center (JPHC)-based Prospective Study**

Huan Song^1,2^, Eiko Saito^2,3,4^, Norie Sawada^3^, Sarah K. Abe^5^, Akihisa Hidaka^3^, Taichi Shimazu^3^, Taiki, Yamaji^3^, Atsushi Goto^3^, Motoki Iwasaki^3^, Shizuka Sasazuki^3^, Weimin Ye^1^, Manami Inoue^2,3*^ and Shoichiro Tsugane^3**^

1. Department of Medical Epidemiology and Biostatistics, Karolinska Institutet, Stockholm, Sweden

2. AXA Department of Health and Human Security, Graduate School of Medicine, The University of Tokyo, 7-3-1 Hongo, Bunkyo-ku, Tokyo, 113-0033, Japan.

3. Epidemiology and Prevention Group, Center for Public Health Sciences, National Cancer Center, 5-1-1 Tsukiji Chuo-ku, Tokyo, 104-0045, Japan.

4. Division of Cancer Statistics Integration, Center for Cancer Control & Information Services, National Cancer Center, 5-1-1 Tsukiji Chuo-ku, Tokyo, 104-0045, Japan.

5. Department of Global Health Policy, Graduate School of Medicine, The University of Tokyo, 7-3-1 Hongo, Bunkyo-ku, Tokyo, 113-0033, Japan

Supplementary Table 1 Associations between perceived stress level and screening-detected cancer incidence, or localized/non-localized cancer incidence at the time of diagnosis

| Perceived stress level | Fully adjusted HR (95% confidence intervals)^*^ | | |
| --- | --- | --- | --- |
|  | Localized cancer | Non-localized cancer | Screening detected cancer |
| *Stress level at baseline* |  |  |  |
| Low | Reference | Reference | Reference |
| Medium | 1.02 (0.94-1.10) | 0.98 (0.93-1.04) | 1.12 (1.00-1.26) |
| High | 1.06 (0.96-1.17) | 0.94 (0.87-1.01) | 1.20 (1.04-1.37) |
| *P for trend* | 0.2318 | 0.1140 | 0.0135 |
| *Dynamic stress level (allowing change of exposure group according to data from 5 year follow-up )* | | | |
| Low | Reference | Reference | Reference |
| Medium | 1.01 (0.95-1.09) | 1.06 (1.00-1.12) | 1.07 (0.97-1.18) |
| High | 1.07 (0.98-1.17) | 1.01 (0.94-1.09) | 1.13 (1.00-1.29) |
| *P for trend* | 0.1731 | 0.5755 | 0.0544 |
| *Long-term stress level* |  |  |  |
| Always low | Reference | Reference | Reference |
| Low or medium (never high) | 1.03 (0.90-1.18) | 0.94 (0.84-1.04) | 1.02 (0.84-1.24) |
| Always medium | 0.99 (0.87-1.13) | 0.97 (0.88-1.07) | 1.12 (0.93-1.34) |
| First high then low/medium | 1.10 (0.95-1.29) | 0.96 (0.85-1.09) | 1.18 (0.96-1.46) |
| First low/medium then high | 1.17 (0.99-1.38) | 1.01 (0.89-1.15) | 1.23 (0.98-1.53) |
| Always high | 1.22 (1.03-1.44) | 1.00 (0.87-1.15) | 1.26 (1.01-1.59) |
| *P for trend* | 0.0016 | 0.4030 | 0.0028 |

^*^Estimated by fully-adjusted Cox proportional hazards regression model (using attained age as underlying time scale), adjusted for sex, psychologically related factors, and other known risk factors for cancer (body mass index, smoking status, alcohol consumption, fruit/vegetable intake, living arrangement, physical activity, occupation, family history of cancer), and stratified by study area.
